# Stereological Study of Changes of GABA-Immunoreactive Neurons in Spinal Dorsal Horn of SNI Rats

**DOI:** 10.1155/2021/6633834

**Published:** 2021-06-18

**Authors:** Hong Liu, Weidong Li, Binbing Xu, Jiduan Jiang, Yuanyuan Zhang, Fan Yang

**Affiliations:** Department of Anesthesiology, Suining Central Hospital, Suining 629000, Sichuan Province, China

## Abstract

**Objective:**

To observe the changes in the mechanical withdrawal threshold (MWT) and the proportion of GABA-immunoreactive neurons in spinal dorsal horn (SDH) of the spared nerve injury (SNI) rat model.

**Methods:**

Thirty-six healthy male SD rats were randomly divided into a sham-operated group (group D, *n* = 18) and an SNI group (group S, *n* = 18). The left sciatic nerve trunk and three branches were exposed, two of which, known as tibial and the peroneal nerve, were ligated and cut off. The sural nerve was preserved to build the SNI model in group S. The left sciatic nerve trunk and three branches were only exposed in group D. MWT tests were performed on the medial and lateral sides of the rats' left hindpaw 1 day before surgery and at 7^th^, 14^th^, and 28^th^ day after surgery.

**Results:**

In group S, compared with the baseline measured 1 day before surgery, MWT on the medial and lateral sides of the rats' left hindpaw decreased significantly on the 7^th^, 14^th^, and 28^th^ days after surgery (*P* < 0.05), while in group D, there was no statistically significant difference (*P* > 0.05). Compared with right SDH, there were not statistically significant reductions in the proportions of GABAergic neurons of left SDH on 7^th^ and 28^th^ day after SNI (*P* > 0.05); however, the proportion of GABAergic neurons in left SDH significantly decreased, compared with that in right side on 14^th^ day after SNI (*P* < 0.05). On the same way, the proportions of GABAergic neurons on 7^th^, 14^th^, and 28^th^ day after surgery were not statistically different (*P* > 0.05) in group D.

**Conclusion:**

The SNI model could reduce the proportion of GABA-immunoreactive neurons in the rat's spinal dorsal horn on the nerve-injured side, and this change was lasting, which might be related to the transformation of the GABA-immunoreactive neurons.

## 1. Introduction

Neuropathic pain (NPP) is a chronic pain caused by nervous system damage or dysfunction and is difficult to treat, with symptoms including spontaneous pain, hyperalgesia, and allodynia. As its mechanism is very complex and the pathogenesis is still unknown, the treatment outcome can be poor, seriously disturbing the patient's emotions and quality of life [1]. Over the past decade, studies on the pathogenesis of NPP have made outstanding progress. Specifically, the function and chemical and structural changes (neuroplasticity) of neurons constitute the characteristic of altered sensitivity to pathological pain; the peripheral sensitization acts on the peripheral receptors, and the central sensitization may occur in the nerve centers of various levels from the spinal dorsal horn (SDH) to the brain [2]. The spared nerve injury (SNI) model is a common and ideal model for study on NPP, with advantages of precise effect of nerve injury, good repeatability, and lasting behavioral changes and can reliably simulate the clinical symptoms of NPP [3]. It is widely believed that the absence of the spinal dorsal horn's inhibition is the key mechanism that causes and maintains NPP. Studies [4, 5] showed that the absent GABAergic inhibition on the lamina I-III of SDH is closely related to NPP, but detailed mechanism remains unknown. By using immunofluorescenct staining, Yowtak [6] found that, 7 days after spinal nerve ligation (SNL) model, the amount of GABA-immunoreactive neurons in the SDH on the operated side was significantly reduced, while the GABA receptor agonists can reverse the rat's hypersensitivity to mechanical stimulation; Inquimbert et al. [7] verified the apoptosis of the spinal GABAergic neurons by using the chronic compression model (CCI) and SNI model of the sciatic nerve. Polgar et al. [8, 9] found the contradictory results: peripheral nerve injury did not cause the loss of the number of GABAergic neurons; some scholars have even found a significantly increased level of spinal GABA after SNL [10]. Therefore, the change in the level of spinal GABA-immunoreactive neurons after peripheral nerve injury and its role in the occurrence of NPP is not yet quite clear. In this study, a stereological morphometric approach was adopted to investigate the three-dimensional changes and changing trend of GABA-immunoreactive neurons in the spinal dorsal horn of SNI rat and to explore the pathogenesis of NPP.

## 2. Materials and Methods

### 2.1. Disclaimer

This experiment was in accordance with all the regulations of animal experiment ethics, and all the laboratory personnel involved were in possession of the qualification certificate of animal experiment.

### 2.2. Animal Grouping

Provided by the Laboratory Animal Center of North Sichuan Medical College (NanChong, China), 36 healthy male SD rats, weighing 260 to 310 g, were randomly divided into a sham surgery group (group D, *n* = 18) and an SNI group (group S, *n* = 18). Both groups were further divided into a 7-day subgroup, a 14-day subgroup, and a 28-day subgroup according to the sampling time, with 6 rats in each subgroup.

### 2.3. Reagents and Instruments

These were the tools used: GABA polyclonal antibody (ab8891, Abcam, UK), immunohistochemistry pen (CIRISC, Japan), SABC immunohistochemical staining kit, DAB color kit (Boster Biological Technology, Ltd., Wuhan, China), Leica slicer (RM 2235, Leica, Germany); dynamic plantar aesthesiometer (37450, Ugo Basile, Italy), culture inverted microscope (TS100-F, Nikon, Japan), BX51 optical microscope system (OLYMPUS, Japan), and Visopharm stereology image system (Denmark).

### 2.4. Modeling

3% pentobarbital sodium was prepared to anesthetize the rats in each group at 50 mg/kg. In group S, after anesthesia, rats were fixed in the prone position on the board, with the skin of the surgical sites exposed by shearing, disinfected with povidone iodine and covered with sterile gauze. The three branches of the sciatic nerve—tibial nerve, common peroneal nerve, and sural nerve—were fully exposed and isolated, the tibial nerve and common peroneal nerve were ligated with 3-0 silk braided medical line and cut off at the distal end, and the sural nerve was retained intact. Then, before the layered suture of the muscle and skin was performed, penicillin powder had been applied at 300,000 units/kg onto the wound, and intramuscular injection of penicillin had been administrated at 200,000 units/kg in the right hind leg to prevent infection after surgery. In group D, after anesthesia, the three branches were exposed without any treatment. Similarly, the layered suture of the muscle and skin and external application and intramuscular injection of penicillin were performed.

### 2.5. Ethological Observation

The rats in each group were weighed before modeling and before sampling, respectively, as indicators evaluating their health and growth status, and the changes in general conditions of the rats in each group after surgery were simultaneously recorded. During the experiment, the rats were placed in the rectangular transparent box of the dynamic plantar esthesiometer. The changes in their activities, gaits, and hindpaw forms were observed, and the ethology of the rats was evaluated as follows: level 1: normal activity, with no deformity in the hindpaw; level 2: normal activity, with obvious deformities (contracture) in the hindpaw; level 3: slightly abnormal activity, with foot drop; and level 4: seriously abnormal activity, with paralyzed hind leg the surgery side.

### 2.6. Mechanical Withdrawal Threshold (MWT) Test

1 day before surgery and on the 7th, 14th, and 28th days after surgery, the “Von Frey” mechanical paw withdrawal threshold was measured with the dynamic plantar esthesiometer on the medial side (saphenous nerve area) and the lateral side (sural nerve area) of the left hindpaw of all rats in each group, with the value measured 1 day before surgery as the baseline. All measurements were taken between 14 : 00 and 16 : 00. Specific measurement methods: after the rat had been adapted to the environment for 15 min, a series of upward forces were applied with the Von Frey filaments onto the medial and the lateral sides of the rat's hindpaw. The upward forces of the filaments were gradually increasing until the hindpaw showed a sharp withdrawal, and the readings on the digital display of the adjuster at this moment were recorded. The values of at least 3 positive responses to the 5 times of stimulation were determined as the rat's MWT, with an interval of at least 20 s between each two tests.

### 2.7. Tissue Sampling

After the MWT had been measured at the corresponding time points (on the 7^th^/14^th^/28^th^ days), the rats in each subgroup were sacrificed and sampled (*n* = 6): intraperitoneal injection of 3% sodium pentobarbital was applied (50 mg·kg-1) to anesthetize the rats. Cardiac perfusion with 200 ml normal saline was first performed, and then approximately 300 ml of 4% paraformaldehyde fixative was perfused from quickly to slowly for fixation. After that, the lumbar spinal cord segments (L5) of about 2 mm in length were taken and placed into 4% paraformaldehyde for fixation. The samples were dehydrated with a series of graded ethanol, clarified with xylene, embedded in paraffin, and sliced into sections.

### 2.8. Immunohistochemistry

The sections were routinely deparaffinized through graded ethanol to water and incubated in 3% H_2_O_2_ at room temperature for 10 min. After antigen retrieval by heating, the samples were incubated in 5% BSA blocking buffer at room temperature for 20 min, added with drops of GABA polyclonal antibody (1: 500), and incubated at 4°C overnight; on the next day, the samples were added with drops of biotinylated goat anti-rabbit IgG and incubated at 37°C for 20 min. After rinsing with 0.01 M PBS, the samples were added with 1 to 2 drops of reagent SABC (StreptAvidin Biotin-peroxidase Complex) and incubated at 37°C for 20 min. Finally, the samples were added with drops of DAB at room temperature for color developing for 5 min, counterstained with hematoxylin for 2 min, and separated with 0.5% ethanol hydrochloride, before routine dehydration, clarification, and mounting in neutral gum.

### 2.9. Quantitative Analysis of Stereological Morphometry

**Figure 1:**
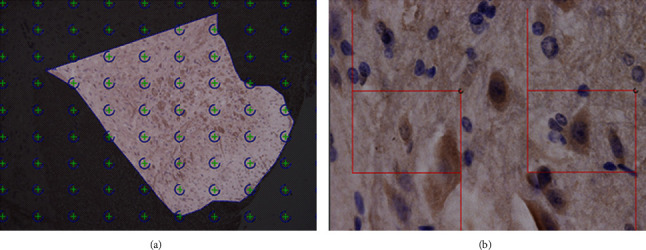
Stereological measurement of the spinal dorsal horn. Note: (a) shows that 10 × 10 dots were superimposed at ×100 magnification objective lens within the outlined range of the spinal dorsal horn, and there were 29 measuring points in the right spinal dorsal horn. (b) shows that 2 optical dissectors of 55.00 *μ*m × 41.41 *μ*m were superimposed at ×1000 magnification objective lens, counting the number of neurons within the dissector.

Visopharm stereology image system and Olympus BX51 Optical Microscope System were employed to observe the sections (See Figure 1). The spinal dorsal horns on both sides were outlined, respectively, in each section with layer drawing, 10 × 10 dots were superimposed at ×10 magnification objective lens with a dot area of 24020.99 *μ*m^2^ (on the lamina I-III of SDH), and the number of dots within the outlined range was counted (*N*). Then, 2 optical disectors were superimposed at ×100 magnification objective lens, with the upper left corner of each dissector as a measuring point. The area of the dissector frame was 55.00 *μ*m × 41.41 *μ*m = 2211.6 *μ*m^2^. The sampling fraction was set at 7%, the focus adjustment was started from 1 *μ*m below the surface of the section, and the number of clearly focused cells of 5 *μ*m in depth within the dissector was counted. Identifying standards for the positive and negative neurons, GABA-immunoreactive neurons had large and round nucleus, with prominent nucleoli, and brownish yellow or yellowish-brown GABA neurotransmitters in the cytoplasm; negative neurons are large and round nucleus, with prominent nucleoli, but their cytoplasm was not colored. The rests were glial cells. The numbers of GABA-immunoreactive neurons and negative neurons in each section were, respectively, counted according to the above method, and the proportion of the number of GABA-immunoreactive neurons on the surgery and the nonsurgery sides of the spinal dorsal horn in the total number of neurons was estimated (*P*_GABA_).

### 2.10. Statistical Analysis

SPSS18.0 software was used for analysis, with all measurement data expressed as mean ± standard deviation (*x* ± *s*). A rank-sum test of two independent samples was adopted to compare the ratings data. A paired *t*-test was adopted for the paired measurement data, and an independent sample *t*-test was adopted to compare two independent samples. *P* < 0.05 was considered statistically significant.

## 3. Results

### 3.1. Weight Comparison of Rats in Each Group

The rats in each group showed no significant difference in body weight before surgery (the test group: 287.90 ± 14.60 g, the control group: 283.38 ± 13.15 g, *P* > 0.05). The weight of rats in each group increased normally at each time points from after surgery to before sampling, and there was no statistically significant difference between the two groups at the same time point (*P* > 0.05).

### 3.2. Ethological Observation

Before surgery, the body size and gait of all rats were normal, with normal shape of the left hindpaw and no abnormal reactions and activities. Rats in group S showed valgus deformity in the hindpaw on the surgery side (the left side), as well as contracture and spontaneous paw withdrawal (95%). The ratings of 4 rats reached level 3, and the other rats were level 2. Compared between before and after surgery, rats in group D showed no obvious abnormalities in activities, gaits, and hindpaw forms, and the ratings of all rats were level 1. There was a statistically significant difference between the two groups (*P* < 0.05) (see Table 1).

### 3.3. Changes in MWT

In group S, MWT on the medial and lateral sides of the rats' left hindpaw decreased significantly. In group S, compared with the baseline, MWT on the medial and lateral sides of the rats' left hindpaw decreased significantly on the 7^th^, 14^th^, and 28^th^ days after surgery (*P* < 0.05), and the lateral MWT decreased more significantly than the medial (*P* < 0.05); while in group D, compared with the baseline before surgery, the medial and lateral MWT of the rats' left hindpaw showed no statistically significant difference at any time point after surgery (*P* >0.05), see Tables 2 and 3 for details.

### 3.4. Immunohistochemical Staining and Stereological Measurement

**Figure 2:**
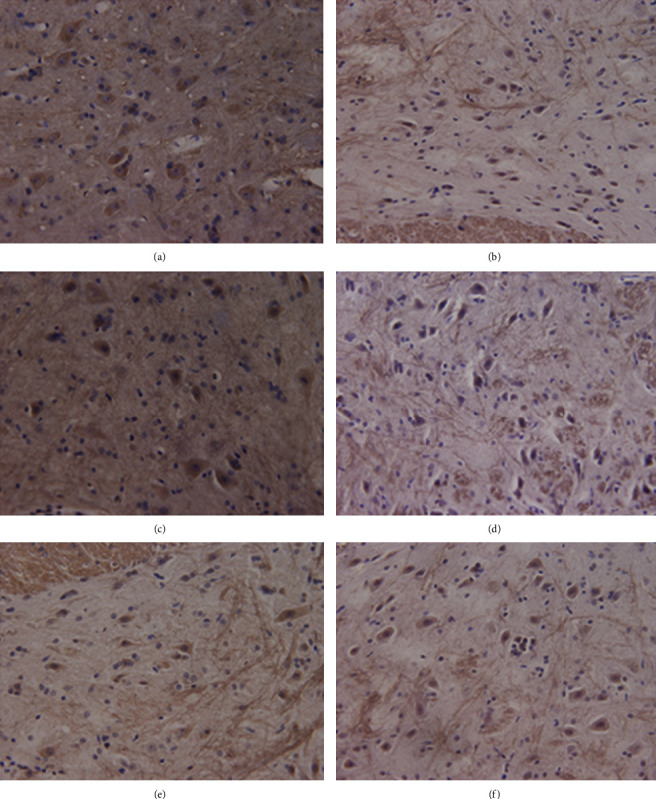
Immunohistochemical staining of spinal dorsal horn of rats in two groups (×400). Note: this figure shows the results of immunohistochemical staining of spinal dorsal horn of rats in groups S and D at each time point: (a) Group S, day 7. (b) Group D, day 7. (c) Group S, day 14. (d) Group D, day 14, (e) Group S, day 28. (f) Group D, day 28. On the 14^th^ day after surgery, the proportion of GABAergic neurons in the spinal dorsal horn in group S was decreased more significantly than that in group D (*P* < 0.01), while there was no significant difference between the two groups on the 7th and 28th days after surgery (*P* > 0.05).

**Figure 3:**
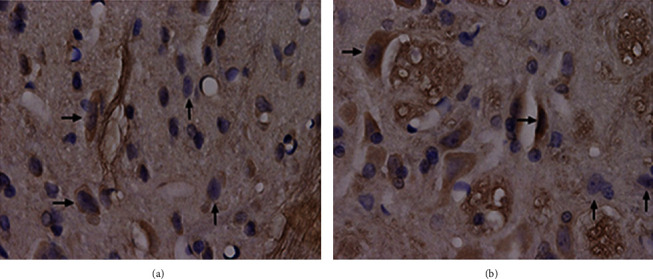
Immunohistochemical staining of the left and right spinal dorsal horns of rats in group S on the 14^th^ day after surgery (×1000). Note: this figure shows the L5 spinal dorsal horn sections of rats in groups S (a) and D (b) on the 14th day after surgery. → represents the immunohistochemical staining positive neuron, and ↑ represents the negative neuron.

**Figure 4:**
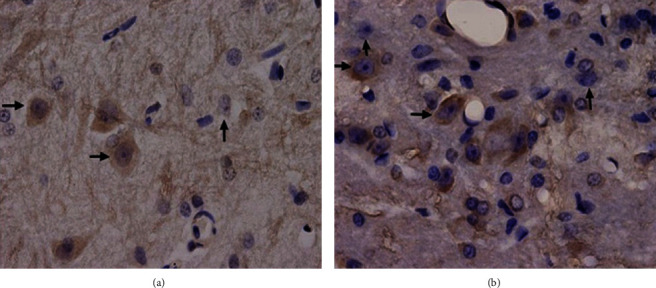
Immunohistochemical staining of the spinal dorsal horn of rats in groups S and D on the 14^th^ day after surgery (×1000). Note: this figure shows the L5 spinal dorsal horn sections of rats in groups S (a) and D (b) on the 14th day after surgery. → represents the immunohistochemical staining positive neuron, and ↑ represents the negative neuron.

**Figure 5:**
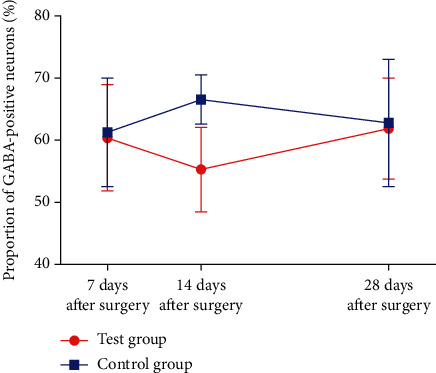
The proportion of GABA-immunoreactive neurons in the left spinal dorsal horn of rats in the two groups.

**Figure 6:**
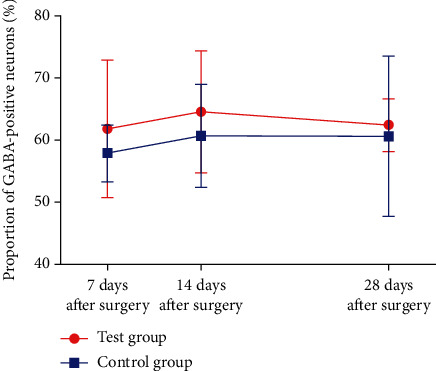
The proportion of GABA-immunoreactive neurons in the right spinal dorsal horn of rats in the two groups.

Results of GABA immunohistochemical staining of the rats in two groups were shown in Figures 2–4. Results of stereological measurement showed that, in group S, *P*_GABA_ on the left side (the surgery side) was reduced slightly more than the right side (the nonsurgery side) on the 7th and 28th days after surgery, but the difference was not statistically significant (*P* > 0.05), while *P*_GABA_ on the left side was reduced more than the right side on the 14^th^ day, with a statistically significant difference (*P* < 0.05); while in group D, there was no statistically significant difference in *P*_GABA_ between the left and right spinal dorsal horn on the 7th, 14th, and 28th days after surgery (*P* > 0.05). In addition, comparison of spinal dorsal horns on the same sides showed that, in group S, *P*_GABA_ on the left side was reduced slightly more than the right side on the 7th and 28th days after surgery, but the difference was not statistically significant (*P* > 0.05), while *P*_GABA_ on the left side in group S was reduced more significantly than the control group on the 14th day after surgery, with a statistically significant difference (*P* < 0.01) (Table 4, Figures 5 and 6).

## 4. Discussion

In this study, after surgery, SNI rats were found with valgus deformity, spontaneous paw withdrawal, and mechanical allodynia, which continued throughout the observation period, and no significant difference in MWT was found in the sham surgery group during the whole process. MWT on the medial and lateral sides of the SNI rat's surgery side hindpaw continued to decline after surgery, and there was a statistically significant difference between each time points after surgery and the baseline before surgery. For the sham surgery group, MWT on the medial and lateral sides of the rat's surgery side hindpaw showed no statistically significant difference between each time points after surgery and the baseline before surgery. The results were consistent with the SNI model built by Woolf, indicating the successful establishment of the SNI rat model in this study.

The results of this study showed that, through comparison of spinal dorsal horns on the surgery and nonsurgery sides in the SNI group, *P*_GABA_ in the spinal dorsal horn was reduced on the 7th and 28th days after surgery, but the difference was not statistically significant (*P* > 0.05), while *P*_GABA_ on the surgery side was reduced more significantly than the nonsurgery side on the 14^th^ day after surgery, with a statistically significant difference (*P* < 0.05); through comparison of spinal dorsal horns on the same sides, on the 7th and 28th days after surgery, *P*_GABA_ in the spinal dorsal horn on the surgery side in group S was decreased slightly more than in group D, with no statistically significant difference, while on the 14th after surgery, *P*_GABA_ in the left spinal dorsal horn in group S was decreased more significantly than that in group D, with a statistically significant difference (*P* < 0.01); there was no statistically significant difference between the subgroups in group D. On the surface, the results were not exactly consistent with the above studies; however, through indepth analysis of their findings, we found that the study of Mary [11] showed that the GABAergic neurons in CCI rats started to decrease from 3 days after surgery on both the surgery and nonsurgery sides, with a minimum in 2 weeks after surgery, while the number of GABAergic neurons rose again on both sides in 8 weeks after surgery, and the nonsurgery side basically recovered to normal. Study of Ibuki et al. [12] showed that the proportion of GABA-immunoreactive cells on the surgery side in CCI rats was significantly reduced 7 days after surgery, with a minimum level in 2 weeks after surgery. When this loss of GABA neurons continued to 4 or 5 weeks after surgery, the number of GABA neurons on the surgery side would start to rise again, with a significantly increase observed after 7 weeks, though it did not recover to the normal level in the end. We found that the changes in GABA-immunoreactive neurons in this study shared a similar trend with those in the studies of Mary and others, and the different changes at the corresponding time points might be related to the use of a different type of the NPP model.

The results of this study suggested that *P*_GABA_ in the spinal dorsal horn in SNI rats on the surgery side showed a process of dropping (day 14) and rising again (day 28), probably due to the GABA-immunoreactive neurons' own transformation. Ibuki [12] speculated that the decrease of GABAergic neurons in CCI rat after 1 week might be due to the downregulation of the synthesis of GABA neurotransmitters. Mary [11] found that the positive cells of the synthetase of GABA neurotransmitter, glutamic acid decarboxylase (GAD), started to decrease from the 3^rd^ day after surgery and rose again after 1 week, reaching a normal value in 8 weeks. This suggested that the GABA neurotransmitters were reduced in CCI rats' GABAergic neurons; therefore, the GABA level in the cytoplasm was lower than the detection threshold in their methods, manifesting as that the originally GABA positive neurons were converted into “negative.” However, as the positive feedback regulation signal, the low level of GABA neurotransmitter stimulates the expression of GABA synthetase GAD. With the increased expression of GAD, the synthesis of GABA neurotransmitter in the GABAergic neurons increased, and the number of GABAergic neurons also showed a growing trend, showing a “self-healing” state of neuron. In addition, Polgar et al. [13] pointed out that some glycine neurons in the spinal cord exhibited a GABA-immunoreactive character. In this study, since the number of GABA-immunoreactive neurons underwent a process of dropping and rising again within a short period, and it was difficult for the neurons to regenerate themselves, we speculated that the transformation of neurons might occur during the decreasing of GABA-immunoreactive neurons. That is, the neurons which had been originally GABA glycine immune double positive were temporarily transformed into glycine immune single positive neurons during the neuropathic pain, and the abovementioned loss of GABAergic neurons resulted from the apoptosis of GABAergic neurons was not necessary. Because of the changes in the number of GABA neurotransmitters, the function of GABAergic neurons might also be altered during the transformation, which might be related to the decline of SNI rat's MWT and the occurrence of NPP.

As the research and measurement methods in previous studies were summarized, most of them directly adopted the two-dimensional approaches, such as a direct microscopic count, with the presence of human bias and error. In this study, however, a three-dimensional quantitative approach of stereological morphometry [14] that had been recognized as more reliable was employed. By superimposing appropriate stereological probes at the area of the measured section, with the proportion as the indicator to estimate the changes in the number of GABA-immunoreactive neurons, the influences of the differences in the volume of the tissues between before and after being paraffin-embedded were excluded, making the results more accurate and reliable.

## 5. Conclusion

In summary, the decreased *P*_GABA_ in the spinal dorsal horn caused by the SNI rat model might be related to the temporary transformation of GABA-immunoreactive neurons resulted from the downregulation of GABA neurotransmitter. However, the dependence between the specific number of the molecules involved in the downregulation of GABA neurotransmitter and the immune response of the GABA-immunoreactive neurons had not been reported in the past. Moreover, the mutually “quantitative and time-dependent” relationship between the downregulation of GABA neurotransmitter and the consequent upregulation of GAD (GABA synthetase) remains to be further studied.

## Figures and Tables

**Table 1:** Ethological ratings of the rats in two groups (*x* ± *s*).

Group	Number	Level 1	Level 2	Level 3	Level 4
Group S	18	0	14	4	0
Group D	18	18	0	0	0

Note: comparison of the two groups, ^∗^*P* < 0.05.

**Table 2:** The lateral MWT of the rats' left hindpaw in two groups (*x* ± *s*).

Group	1 day before surgery	1 day after surgery	3 days after surgery	7 days after surgery	14 days after surgery	28 days after surgery
	(*n* = 36)	(*n* = 36)	(*n* = 36)	(*n* = 36)	(*n* = 24)	(*n* = 12)
Group S	36.20 ± 4.40	34.00 ± 5.80	35.47 ± 6.78	26.89 ± 8.09^∗^^**#**^	29.54 ± 8.37^∗^^**#**^	24.5 ± 11.98^∗^^**#**^
Group D	35.85 ± 3.96	36.17 ± 7.16	35.64 ± 5.85	34.17 ± 9.51	36.80 ± 7.45	39.00 ± 3.52

Note: Compared with the baseline before surgery, ^∗^*P* < 0.05; compared with group D, ^**#**^*P* < 0.05.

**Table 3:** The medial MWT of the rats' left hindpaw in two groups (*x* ± *s*).

Group	1 day before surgery	1 day after surgery	3 days after surgery	7 days after surgery	14 days after surgery	28 days after surgery
	(*n* = 36)	(*n* = 36)	(*n* = 36)	(*n* = 36)	(*n* = 24)	(*n* = 12)
Group S	38.50 ± 4.20	32.42 ± 5.95^∗^	35.20 ± 7.32	28.56 ± 8.48^∗^^**#**^	29.38 ± 7.50^∗^^**#**^	21.43 ± 11.65^∗^^**#**^
Group D	36.31 ± 4.46	34.67 ± 7.00	37.17 ± 6.69	36.00 ± 10.60	35.20 ± 8.80	35.67 ± 5.99

Note: compared with the baseline before surgery, ^∗^*P* < 0.05; compared with group D, ^**#**^*P* < 0.05.

**Table 4:** The proportion of GABA-immunoreactive neurons in the spinal dorsal horn of rats in two groups (*x* ± *s*).

Group	7 days after surgery (*n* = 12)		14 days after surgery (*n* = 12)		28 days after surgery (*n* = 12)	
	On the left side	On the right side	On the left side	On the right side	On the left side	On the right side
Group S (%)	60.41 ± 8.49	61.94 ± 11.04	55.27 ± 6.80^∗^	64.68 ± 9.77	61.87 ± 8.12	62.58 ± 4.25
Group D (%)	61.27 ± 8.73	58.02 ± 4.58	66.59 ± 3.97	60.84 ± 8.28	62.81 ± 10.26	60.74 ± 12.88

Note: compared with the right side, ^∗^*P* < 0.05; compared with group D, ^∗^*P* < 0.01.

## Data Availability

All data has been incorporated into this article.
